# Efficiency of pragmatic search strategies to update clinical guidelines recommendations

**DOI:** 10.1186/s12874-015-0058-2

**Published:** 2015-07-31

**Authors:** L. Martínez García, AJ. Sanabria, I. Araya, J. Lawson, I. Solà, RWM. Vernooij, D. López, E. García Álvarez, MM. Trujillo-Martín, I. Etxeandia-Ikobaltzeta, A. Kotzeva, D. Rigau, A. Louro-González, L. Barajas-Nava, P. Díaz del Campo, MD. Estrada, J. Gracia, F. Salcedo-Fernandez, RB. Haynes, P. Alonso-Coello, Updating Guidelines Working Group

**Affiliations:** Iberoamerican Cochrane Centre - Biomedical Research Institute Sant Pau (IIB Sant Pau), Barcelona, Spain; Evidence Based Dentistry Unit, Faculty of Dentistry, Universidad de Chile, Santiago, Chile; Department of Clinical Epidemiology and Biostatistics, McMaster University, Hamilton, Canada; Department of Epidemiology, Sub Secretariat of Public Health, Ministry of Health, Santiago, Chile; NHS Ayrshire and Arran, Ayr, UK; Fundación Canaria de Investigación y Salud (FUNCIS), Red de Investigación en Servicios de Salud en Enfermedades Crónicas (REDISSEC), Tenerife, Spain; Osteba, Basque Office for Health Technology Assessment, Vitoria, Spain; Agency for Health Quality and Assessment of Catalonia (AQuAS), Barcelona, Spain; CIBER of Epidemiology and Public Health (CIBERESP), Barcelona, Spain; Centro de Saúde de Cambre, Xerencia de Xestión Integrada de A Coruña SERGAS, A Coruña, Spain; Health Technology Assessment Unit (UETS), Subdirección General de Tecnología e Innovación Sanitaria, Consejería de Sanidad, Madrid, Spain; National Clinical Practice Guideline Programme of the NHS, Madrid, Spain; GuíaSalud-Aragon Institute of Health Sciences, Zaragoza, Spain

**Keywords:** Clinical guidelines, Diffusion of innovation, Dissemination and implementation, Evidence-based medicine, Information storage and retrieval, Knowledge translation, Methods, Updating

## Abstract

**Background:**

A major challenge in updating clinical guidelines is to efficiently identify new, relevant evidence. We evaluated the efficiency and feasibility of two new approaches: the development of restrictive search strategies using PubMed Clinical Queries for MEDLINE and the use of the PLUS (McMaster Premium Literature Service) database.

**Methods:**

We evaluated a random sample of recommendations from a national guideline development program and identified the references that would potentially trigger an update (key references) using an exhaustive approach.

We designed restrictive search strategies using the minimum number of Medical Subject Headings (MeSH) terms and text words required from the original exhaustive search strategies and applying broad and narrow filters. We developed PLUS search strategies, matching Medical Subject Headings (MeSH) and Systematized Nomenclature of Medicine (SNOMED) terms with guideline topics. We compared the number of key references retrieved by these approaches with those retrieved by the exhaustive approach.

**Results:**

The restrictive approach retrieved 68.1 % fewer references than the exhaustive approach (12,486 versus 39,136), and identified 89.9 % (62/69) of key references and 88 % (22/25) of recommendation updates. The use of PLUS retrieved 88.5 % fewer references than the exhaustive approach (4,486 versus 39,136) and identified substantially fewer key references (18/69, 26.1 %) and fewer recommendation updates (10/25, 40 %).

**Conclusions:**

The proposed restrictive approach is a highly efficient and feasible method to identify new evidence that triggers a recommendation update. Searching only in the PLUS database proved to be a suboptimal approach and suggests the need for topic-specific tailoring.

**Electronic supplementary material:**

The online version of this article (doi:10.1186/s12874-015-0058-2) contains supplementary material, which is available to authorized users.

## Background

Clinical guidelines, like systematic reviews and other evidence summaries, require periodic reassessment of research evidence to remain valid [1–4]. Current guidance usually recommends revision and update within two to three years of their publication [5, 6]. New evidence to update clinical guidelines is generally identified using the original exhaustive search strategies [7].

A major challenge for guideline developers is to efficiently screen for new, relevant evidence that justifies a clinical guideline update. Unfortunately, little empirical work has been conducted to date to test the effectiveness and efficiency of searching processes [7]. More than a decade ago, Shekelle et al. developed a strategy based on retrieving reviews, editorials, and commentaries in high impact general journals and specialised journals, complemented with a survey by clinical experts [8]. Gartlehner et al. compared a modified version of this strategy versus an exhaustive search strategy [9]. The results so far have shown that restrictive approaches are promising, but more information is needed about the timing and type of search [7].

Similarly, researchers are testing alternative search strategies to update systematic reviews [10–13]. Haynes et al. developed the McMaster Premium Literature Service (PLUS) database, from the McMaster Health Knowledge Refinery [14, 15]. PLUS contains a searchable subset of pre-appraised primary studies and systematic reviews from more than 120 journals and it can identify key articles needed to update systematic reviews [14, 15]. Clinical Queries search filters in MEDLINE and EMBASE have also shown a high sensitivity to detect key articles [11].

We designed a study to evaluate the efficiency and feasibility of two approaches to identify the need to update clinical guidelines recommendations: 1) restrictive search strategies using PubMed Clinical Queries search filters for MEDLINE and 2) the use of PLUS database.

## Methods

### Design

We conducted a descriptive study of search strategies to identify the references that update recommendations from clinical guidelines. We developed three search strategies to identify the need to update the recommendations: an exhaustive approach, a restrictive approach, and a PLUS approach.

The sample was obtained from a previous study and included a stratified random sample of recommendations from the Spanish National Health System Clinical Guidelines Program [1, 16]. The selection process involved two phases: 1) we stratified guidelines by topic and by year of publication; when multiple guidelines per strata were available, we randomly selected one; 2) we performed a stratified random sampling of recommendations by guideline topic and by turnover (number of pertinent references linked per recommendation in the updating process).

### 1) Exhaustive approach

Guideline methodologists with experience designing search strategies developed exhaustive literature search strategies for each clinical question: 1) based on the original searches; and 2) applying the filters of the original study. An example of the exhaustive search strategy is available in Additional file 1. We also contacted clinical experts to identify new studies. We obtained a reference database of clinical questions. We screened the references and assessed them qualitatively as: 1) *Pertinent references:* Randomised controlled trials or systematic reviews related to the topic of the clinical guideline; 2) *Relevant references:* pertinent references that could be used when considering an update to a recommendation, but that would not necessarily trigger a potential update; and 3) *Key references:* relevant references that would potentially trigger an update because of their impact on the population, the intervention, the comparison, the outcome, the quality of the evidence, the direction and/or the strength of the recommendation. Using the results of the reference screening we classified recommendations as: 1) need for updating*:* with one or more key references linked; or 2) still valid: without key references linked.

A more complete description of this approach is available in the previously published protocol and survival analysis results [1, 16].

### 2) Restrictive approach

**Fig. 1:**
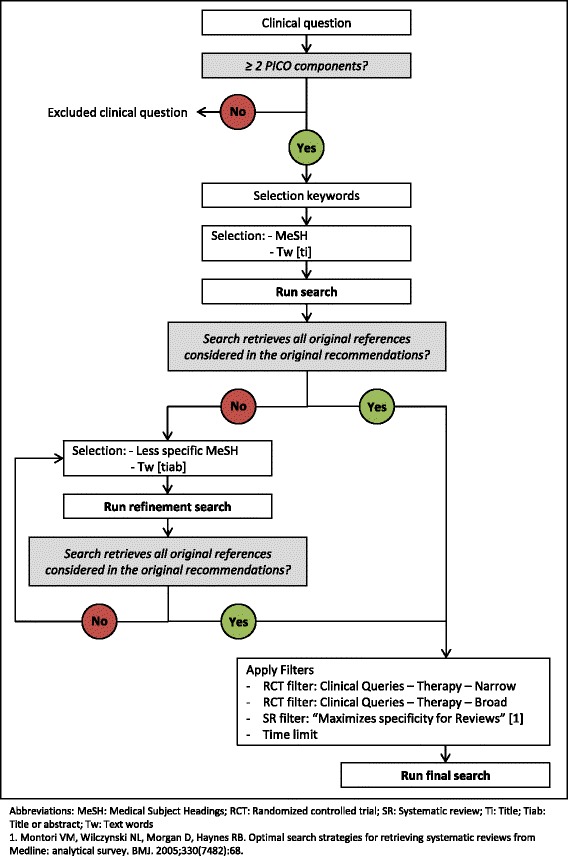
Restrictive approach algorithm. Figure01_RestrictiveApproachAlgorithm. Microsoft PowerPoint presentation

Guideline methodologists, trained by researchers with experience designing search strategies, developed restrictive search strategies for each clinical question using the PubMed Clinical Queries search filters for the MEDLINE database. We considered clinical questions that had at least two PICO (population, intervention, comparator or outcome) components. We developed the restrictive search strategies considering the minimum number of Medical Subject Headings (MeSH) terms and text words required from the original exhaustive searches strategies. The search strategies were designed in four stages [Fig. 1]: 1) Development: we selected keywords from the clinical questions and identified Medical Subject Headings (MeSH) terms and text words in titles; 2) Validation: we evaluated whether each search retrieved all the original references for its corresponding recommendation; 3) Refinement: If a search did not retrieve all the original references, we selected and searched less specific Medical Subject Headings (MeSH) and/or text words in the title or abstract; and 4) Application of each of a broad and a narrow treatment Clinical Queries filter (www.ncbi.nlm.nih.gov/pubmed/clinical), and a systematic review filter [17]. We used the same date limits as with the exhaustive approach (from the complete year in which the original exhaustive searches was completed onwards). An example of a restrictive search strategy is available in Additional file 1.

### 3) PLUS approach

An information specialist from the Health Information Research Unit developed a PLUS search strategy for each guideline topic. We matched Medical Subject Headings (MeSH) and Systematized Nomenclature of Medicine (SNOMED) indexing terms in the PLUS database with clinical guideline topics. Both primary and review papers were included. To take into account the time delay associated with the critical appraisal process (CAP) the articles go through, we ran the PLUS searches strategies from the beginning of the year in which the original exhaustive searches were run, until approximately three months beyond the latest date of the exhaustive searches. An example of a PLUS search strategy is available in Additional file 1.

### Outcome

Our primary outcome was the number of key references identified by each alternative approach.

### Statistical methods

We performed a descriptive analysis of the data. We calculated absolute and relative frequencies or median and range, as appropriate.

**Fig. 2:**
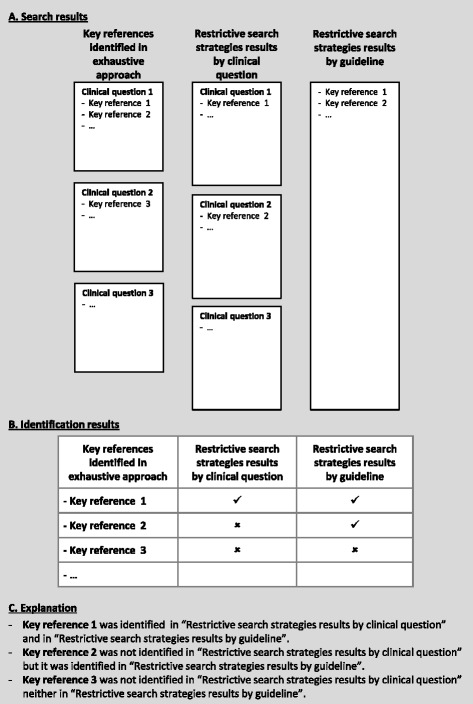
References analysis. Figure02_ReferencesAnalysis. Microsoft PowerPoint presentation

Two investigators independently retrieved the key references (identified in the exhaustive approach) in each of the alternative approach results. We analysed the number of key references in: 1) the results of restrictive search strategies per clinical question; 2) restrictive search strategies results per clinical guideline (clustering all references identified by clinical question) [Fig. 2]; and 3) results of PLUS strategies per clinical guideline. We did not identify additional pertinent, relevant or key references from the alternative approaches. We did not develop restrictive search strategies for clinical questions with less than two of the four PICO components, prognosis or diagnostic clinical questions. In these instances we used the updated exhaustive search strategies.

We identified the recommendations that needed an update (with one or more key references) retrieved by each alternative approach. We compared the recommendations identified with those that were not identified according to clinical guideline topic (cancer, cardiovascular disease, mental health or metabolic disease), strength of recommendation (A, B, C, D or good practice point [18]), clinical purpose (prevention, screening, diagnosis, treatment or other), and turnover. Each recommendation was classified according to the number of linked pertinent references: none, ≤ median number (low turnover), or > median number (high turnover). We used Pearson’s chi-square test or Fisher’s exact test, as appropriate.

We recorded the number of hours spent on designing each approach and the number of researchers involved.

We accepted p values of less than 0.05 as significant in all calculations. We performed the analyses using SPSS 21.0 (SPSS Inc., Chicago, Illinois).

## Results

We included a cohort of four clinical guidelines from the Spanish National Health System Clinical Guidelines Programme, corresponding to 87 clinical questions and 249 recommendations [19–22]. After the random selection process, the final recommendation sample included 43 clinical questions and 113 recommendations.

### Exhaustive approach results

This approach retrieved a total of 39,136 references from the four clinical guidelines included. From the recommendations sample, we identified a total of 69 key references and 25 recommendations that potentially needed an update [Table 1].

**Table 1:** Exhaustive approach results

	Major depression in adults 2008 [19]	Obesity in childhood and adolescence 2009 [20]	Prostate cancer treatment 2008 [21]	Secondary prevention of stroke 2009 [22]	Total
Search period (years)	4.8	3.9	4.5	5.1	
References retrieved in search for clinical guidelines, n	11243	9763	3343	14787	39136
Key references identified from recommendation sample, n	13	32	11	13	69
Potential update recommendations identified from recommendation sample, n (%)	3	8	7	7	25

### Restrictive approach results

We applied the restrictive approach to 88.5 % (77/87) clinical questions from the included clinical guidelines, corresponding to 85 % (96/113) of the recommendations from our recommendation sample. We excluded eight questions that did not present a minimum of two PICO components (population, intervention, comparator or outcome) and one diagnostic question.

The restrictive searches covered a mean of 4.6 years (range 3.9 – 5.1 years) from 2008–2009 to 2011 – 2012 [Table 2].

**Table 2:** Restrictive approach results

	Major depression in adults 2008 [19]		Obesity in childhood and adolescence 2009 [20]		Prostate cancer treatment 2008 [21]		Secondary prevention of stroke 2009 [22]		Total	
Search period (years)	4.8		3.9		4.5		5.1			
References retrieved in search for clinical guidelines, n										
- Broad filter	9223		10561		6939		13294		40017	
- Narrow filter	2814		3976		976		2187		9953	
Key references identified from recommendation sample, n (%)^a^										
- Exhaustive approach^b^	13		16		4		13		46	
- Broad filter										
by individual clinical questions	5	38.5	11	68.8	4	100.0	6	46.2	26	56.5
by clustering all clinical questions	11	84.6	16	100.0	4	100.0	9	69.2	40	87.0
- Narrow filter										
by individual clinical questions	4	30.8	11	68.8	4	100.0	6	46.2	25	54.3
by clustering all clinical questions	10	76.9	16	100.0	4	100.0	9	69.2	39	84.8
Potential update recommendations identified from recommendation sample, n (%)^a^										
- Exhaustive approach^b^	3		6		4		7		20	
- Broad filter										
by individual clinical questions	3	100.0	4	66.7	4	100.0	4	57.1	15	75.0
by clustering all clinical questions	3	100.0	6	100.0	4	100.0	5	71.4	18	90.0
- Narrow filter										
by individual clinical questions	2	66.7	4	66.7	4	100.0	4	57.1	14	70.0
by clustering all clinical questions	2	66.7	6	100.0	4	100.0	5	71.4	17	85.0

^a^Percentage of references and recommendations identified regarding the exhaustive strategy

^b^Exhaustive strategy results without clinical questions and recommendations not included in ReSe strategy

For the clinical guidelines included, we retrieved a total of 40,021 references using the broad filter and 9,958 references using the narrow filter [Table 2]. We retrieved more key references when we clustered results of references per guideline rather than per question (40 [87 %] and 39 [84.8 %] compared with 26 [56.5 %] and 25 [54.3 %] using the broad and narrow filters, respectively) [Table 2, Additional file 2]. Similarly, clustered results of references per guideline identified a higher number of recommendations that were considered to potentially need an update (18 [90.0 %] and 17 [85 %] compared with 15 [75 %] and 14 [70 %] respectively [Table 2].

When we used exhaustive search strategies for the clinical questions not developed by the restrictive approach (narrow filter and clustering by all questions), we retrieved a total of 12,486 references, and we identified a total of 62 (89.9 %) key references and 22 (88.0 %) recommendations that potentially needed an update [Table 4].

**Fig. 3:**
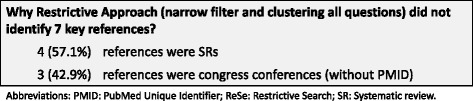
Key references not identified by restrictive approach. Figure03_RefNotIdentifiedRestrictive. Microsoft PowerPoint presentation

The restrictive approach (narrow filter and clustering by all questions) failed to identify seven key references (15.2 %): four (57.1 %) references were systematic reviews and three references (42.9 %) were congress abstracts (not indexed in MEDLINE) [Fig. 3].

The recommendations that potentially needed an update not identified by the restrictive approach were similar to those that were identified in terms of topic, strength of the recommendations, clinical purpose, and turnover [Additional file 3].

### PLUS approach results

The PLUS searches covered a median of 5.0 years (range 4.1 – 5.3 years) from 2008–2009 to 2011 – 2012 [Table 3].

**Table 3:** PLUS approach results

	Major depression in Adults 2008 [19]		Obesity in childhood and adolescence 2009 [20]		Prostate cancer treatment 2008 [21]		Secondary prevention of stroke 2009 [22]		Total	
Search period (years)	5.3		4.1		4.8		5.3			
References retrieved in search for clinical guidelines, n	973		317		137		3059		4486	
Key references identified from recommendation sample, n (%)^a^										
- Exhaustive strategy	13		32		11		13		69	
- PLUS strategy	4	(30.8)	9	(28.1)	1	(9.1)	4	(30.8)	18	(26.1)
Potential update recommendations identified from recommendation sample, n (%)^a^										
- Exhaustive strategy	3		8		7		7		25	
- PLUS strategy	2	(66.7)	4	(50.0)	1	(14.3)	3	(42.9)	10	(40.0)

^a^Percentage of references and recommendations identified regarding the exhaustive strategy

For the clinical guidelines included, we retrieved a total of 4,486 references (range 137 – 3,059) [Table 3]. For the recommendation sample, we retrieved 18 (26.1 %) key references; these references potentially update 10 (40 %) recommendations [Table 3, Additional file 2].

**Fig. 4:**
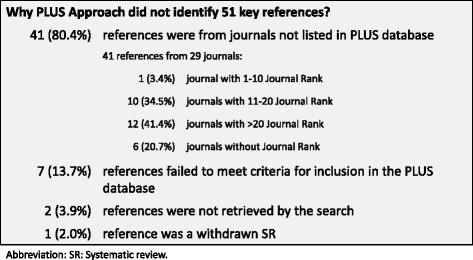
Key references not identified by PLUS approach. Figure04_RefNotIdentifiedPLUS. Microsoft PowerPoint presentation

The PLUS approach failed to identify 51 key references (73.9 %); most (41 references, 80.4 %) were from journals not included in PLUS database [Fig. 4].

Recommendations with a high turnover were more likely to be identified by the PLUS approach. The remaining factors (clinical guideline topic, strength of the recommendations, and clinical purpose) were not significantly associated with the need to update [Additional file 3].

### Resource use

Three guideline methodologists spent a total of 174 h in designing and running the restrictive search strategies [Table 4]. The PLUS search strategies were developed by an information specialist who designed and ran the searches in 28 h [Table 4].

**Table. 4:** Summary results by approach

	Exhaustive approach		Restrictive approach^a^		PLUS approach	
	n	%	n	%^b^	n	%^b^
References identification						
References retrieved in search for clinical guidelines	39136		12486	31.9	4486	11.5
Key references identified from recommendation sample	69		62	89.9	18	26.1
Recommendation identification						
Potential update recommendations identified from recommendation sample	25		22	88.0	10	40.0
Resource use						
Guidelines methodologists	4		3	75.0	-	
Information specialist	-		-		1	25.0
Time to perform the search (hours)	279		174.3	62.5	28	10.0

^a^Narrow filter, clustered by all questions, and imputed exhaustive search results for the clinical questions not included in the restrictive approach

^b^Percentage regarding the exhaustive approach

## Discussion

We evaluated two search strategies to identify signals for updating recommendations and compared them to an exhaustive search strategy using a random sample of recommendations from a cohort of clinical guidelines from a national guideline development program.

The restrictive approach (using a narrow PubMed Clinical Queries filter, clustering results per clinical guideline and imputing exhaustive search results for clinical questions not developed) retrieved 68.1 % fewer references than the exhaustive approach, and identified most of the key references (62/69, 89.9 %) and recommendations updates (22/25, 88.0 %). We developed search strategies for each clinical question but obtained better results by considering the results across all questions included in a clinical guideline. The restrictive approach proved to be relatively simple to develop, not needing the expertise of information retrieval specialists. Over half of the very few missing key references with this approach were systematic reviews. Three references were missed due to a mistake in the design of restrictive searches, and one was missed by the filter used [17], reflecting the need to pay more attention to the design and quality check of search strategies. Additional searches for systematic reviews in specific databases, like Epistemonikos, could prove useful [www.epistemonikos.org/]).

Our results show that PLUS approach retrieved 88.5 % fewer references than the exhaustive approach but identified a substantially lower number of key references (18/69, 26.1 %) and potential updates (10/25, 40 %) than the restrictive approach. These results were similar independently of the searches being performed by a PLUS information specialist (using search strategies) or directly using the PLUS interface using topic synonyms (*post-hoc analysis*). This poor performance was mainly due to most of these key references (80.4 %) being from journals not included in PLUS database.

The PLUS approach performed differently across topics with major depression performing best (66.7 % of key references retrieved) and prostate cancer worst (14.3 %). This poor performance in the prostate cancer guideline is explained by the fact that the PLUS database does not include a large number of urology journals. This resource includes a limited number of journals with a stronger focus on a limited number of specialties and health topics. Given these findings and building on previous research in the systematic reviews and clinical guidelines fields, post-hoc we explored a potential approach of tailoring the PLUS approach by adding a limited number of journals for each specialty (e.g. those with a higher impact factor) [8, 9, 12, 13]. However, missing key references were published in a highly heterogeneous sample of journals, with only 3.4 % being in the first decile [Fig. 4].

The two search strategies we tested were far less time consuming than the exhaustive search strategy. The restrictive approach needs initial tailoring and takes each original guideline, question, search and references into account. In contrast, the PLUS approach could be potentially executed directly in its interface simply using topic synonyms from clinical guidelines.

### Our results in the context of previous research

Only one previous study of clinical guidelines compared a different type of restrictive approach versus an exhaustive approach [9]. However, this study considered prevention topics as the unit of analysis rather than the individual recommendations. Furthermore, the authors restricted the search to MEDLINE, using publication types (review articles, editorials, guidelines and commentaries) and limiting the search to core and specialty clinical journals [9].

A recent evaluation of NICE clinical guidelines for interventional procedures also showed that updated recommendations that required a modification generally had a greater increase in their evidence base (number of patients included in observational studies published) than non-updated recommendations [23]. Our results are consistent with this finding, showing a higher efficiency of the PLUS approach in recommendations with a higher turnover.

There is indirect evidence about the performance of PLUS for clinical guidelines from a previous study that evaluated the updating of systematic reviews [11]. Only 13 out of 87 systematic reviews (14.9 %) included all the new studies in PLUS. In 39 (44.8 %) reviews there was no statistically significant difference between PLUS and non-PLUS new studies (ROR: 0.99; 95 % confidence interval: 0.87-1.14). Thirty-five updated reviews (40.2 %) had no new studies indexed in PLUS (although conclusions were seldom altered by addition of new studies) [11]. Despite these results in systematic reviews, the PLUS database did not perform similarly in the context of clinical guidelines. However, we did not routinely determine the change in effect sizes with key references, so we could not assess their quantitative relationship. Neither did we assess whether references identified in the PLUS database could have reliably signalled the need to update for topics that were in the journals that are included.

The same study by Hemens et al. confirmed the high sensitivity of Clinical Queries filters for MEDLINE and EMBASE in detecting randomized controlled trials [11]. This is consistent with our results showing that incorporating Clinical Queries filters (to identify randomized controlled trials) and Montori’s et al. filter (to identify systematic reviews) significantly reduces the citation screening burden [17].

### Strengths and limitations

We used a rigorous and explicit methodology building on previous research in this area, improving its deficiencies, and implementing an innovative solution. We also used the exhaustive approach as a standard, improving the validity of the results and, hence, the strength of our inferences. We independently screened and extracted the data in pairs and included methodologists and panel members from the original guidelines as far as possible. Finally, we laid out a structured framework (e.g., outcome definitions) that could prove useful in the future for other researchers in the field.

Our study has some limitations. We did not assess all references retrieved by each alternative approach, so we were not able to evaluate whether other key references were identified by any of these approaches. Our sample is limited to recommendations from four guidelines topics. However, this potential limitation is mitigated because our sample covers broad areas such as cancer, cardiovascular diseases, mental health and lifestyle and behavioural issues. Additionally, we based our exhaustive search strategies on searches specifically designed during the original guidelines development. A post-hoc analysis revealed several mistakes and inconsistencies in search strategies that could have been avoided through peer review process [24]. However, the validation of the accuracy of the original search strategies was beyond the scope of our study. We are unable to estimate how this issue could affect the recall of the exhaustive search strategies, although we think that these deficiencies are minor and that they do not alter our conclusions. We included only randomised controlled trials and systematic reviews and did not incorporate observational studies, diagnostic questions or evidence about values and preferences or resource use considerations. Finally, some authors had conflicts of interest due to their involvement in the PLUS database and Clinical Queries filter development. However, they did not participate in the identification of key references.

## Conclusions

Our results have important implications both for the updating of guidelines and for future research in this field. The proposed method of developing restrictive search strategies, using PubMed Clinical Queries filters in the MEDLINE database, provides a feasible and efficient method for guideline developers to identify significant new studies that are likely to trigger a recommendation update. Searching only in the PLUS database was a suboptimal approach that needs topic specific tailoring.

Our results highlight the need for additional methodological research in this field. For this future work, investigators are likely to find our framework helpful.

## Additional files

Additional file 1:
**Search strategies examples.** We reported an example of exhaustive strategy, restrictive strategy, PLUS strategy. (PDF 82 kb) Additional file 2:
**Key references by approach.** We reported key references by strategy, according to their clinical guidelines and linked to recommendation. (PDF 62 kb) Additional file 3:
**Additional tables.** We reported complementary results (PDF 40 kb)
