# Supplementary material for: Efficiency of pragmatic search strategies to update clinical guidelines recommendations
Source: BMC Med Res Methodol. 2015 Jul 31;15:57. doi: 10.1186/s12874-015-0058-2 (PMC4521498; doi:10.1186/s12874-015-0058-2)
Supplement: Additional file 1: — Search strategies examples. We reported an example of exhaustive strategy, restrictive strategy, PLUS strategy. (PDF 82 kb) [file 12874_2015_58_MOESM1_ESM.pdf]

Additional file 1: Strategies examples

**Clinical guideline**<br>Clinical Practice Guideline on the Management of Major Depression in Adults, 2008
**Clinical question**<br>Is treatment with St John's Wort effective?

1. *Exhaustive approach*

1.1. Randomized control trials

1.1.1. MEDLINE

5	Search #3 AND #4
4	Search ("Clinical trials as Topic"[MH] OR "clinical trial"[PT] OR "controlled clinical trial"[PT] OR "single blind method"[MH] OR "double blind method"[MH] OR "cross over studies"[MH] OR "random allocation"[MH]) OR ((Trial*[TIAB] OR Random*[TIAB]) AND ("blind"[TIAB]))
3	Search #1 AND #2 Limits: English, French, Italian, Spanish, Catalan, Portuguese, All Adult: 19+ years, Publication Date from 2007 to 2011
2	("Depressive Disorder"[MH] OR "Depressive Disorder, Major"[MH] OR "Dysthymic Disorder"[MH] OR "Seasonal Affective Disorder"[MH] OR Depressi*[TI] OR Dysthym*[TI] OR Seasonal[TI] OR "Affective"[TI]) NOT ("Bipolar Disorder"[MH] OR "Depression, Postpartum"[MH] OR Mania*[TI] OR Bipolar*[TI] OR Puerper*[TI] OR Postpart*[TI] OR Pregnan*[TI] OR Post-Natal*[TI] OR Postnatal[TI]) Limits: English, French, Italian, Spanish, Catalan, Portuguese, All Adult: 19+ years, Publication Date from 2007 to 2011
1	"Hypericum"[MeSH] OR "Hypericum perforatum" [TW] OR "Saint John's Wort" [TW] OR "St. John's Wort" [TW] OR "St. Johns Wort" [TW]

1.1.2. EMBASE

1	exp 'hypericum perforatum'/
2	("Hypericum" or "Hypericum perforatum" or "Saint John's Wort" or "St. John's Wort" or "St. Johns Wort").ti,sh,hw,ab,tn,dm,kw.
3	1 or 2
4	exp depression/ or exp recurrent brief depression/ or exp endogenous depression/ or exp reactive depression/ or exp major depression/ or exp involutional depression/
5	exp dysthymia/
6	exp seasonal affective disorder/
7	4 or 5 or 6
8	(depressi* or dysthym* or "Seasonal Affective" or Involutional*).ti.

9	exp manic psychosis/
10	exp bipolar disorder/
11	puerperal depression/
12	9 or 10 or 11
13	(Mania* or Bipolar* or Postpart* or Puerper* or Pregnant*).ti.
14	7 or 8
15	12 or 13
16	14 not 15
17	limit 16 to (embase and (basque or catalan or english or french or gallegan or italian or portuguese or spanish) and yr="2006 - 2011" and adult <18 to 64 years>)
18	exp clinical trial/
19	exp crossover procedure/
20	exp randomization/
21	exp randomized controlled trial/
22	exp double blind procedure/
23	exp single blind procedure/
24	exp triple blind procedure/
25	(single or double or triple).ti,ab.
26	(blind and method*).ti,ab.
27	25 and 26
28	"random*".ti,ab.
29	"trial*".ti,ab.
30	18 or 19 or 20 or 21 or 22 or 23 or 24 or 27 or 28 or 29
31	"systematic review".af,ti,ab.
32	30 not 31
33	17 and 32
34	3 and 33

1.1.3. Psychinfo

1	exp hypericum perforatum/
2	("Hypericum" or "Hypericum perforatum" or "Saint John's Wort" or "St. John's Wort" or "St. Johns Wort").ab,hw,id,sh,ti.
3	1 or 2
4	clinical trials/
5	(randomized adj4 Trials).ab,ti.
6	(crossover adj5 design).ab,ti.
7	(random adj4 assignment).ab,ti.
8	(double adj1 blind).ab,ti.
9	(single adj1 blind).ab,ti.
10	(triple adj1 blind).ab,ti.
11	randomization.ab,ti.
12	4 or 5 or 6 or 7 or 8 or 9 or 10 or 11
13	(systematic adj3 review).ab,sh,ti.
14	12 not 13

15	seasonal affective disorder/
16	"depression (emotion)"/
17	reactive depression/
18	recurrent depression/
19	endogenous depression/
20	anaclitic depression/
21	15 or 16 or 17 or 18 or 19 or 20
22	"depress*".ti.
23	seasonal affective disorder.ti.
24	22 or 23
25	21 or 24
26	bipolar disorder/
27	postpartum depression/
28	pregnancy/
29	(mania* or bipolar* or pregnan* or postpart* or puerper*).ti.
30	26 or 27 or 28 or 29
31	25 not 30
32	limit 31 to (adulthood <18+ years> and "300 adulthood " and (catalan or english or french or italian or portuguese or spanish) and yr="2007 - 2011")
33	14 and 32
34	3 and 33

1.2. Systematic reviews

1.2.1. MEDLINE

5	Search #3 AND #4
4	((Systematic Review*[TIAB] OR systematic literature review*[TIAB] OR meta-analysis[PT] OR "meta analysis as topic"[MH] OR meta-analysis[TI] OR meta-analysis[TI] OR metaanalys*[TI] OR "evidence-based"[TI]) OR (("evidence-based" OR Review* OR Overview* OR Survey* OR recommendation* OR consensus development conference[PT] OR health planning guidelines OR guideline[PT]) AND (systematic[TIAB] OR systematically OR critical[TIAB] OR studie*[TIAB] OR study[TIAB] AND selection[TIAB] OR (inclusion criteri*) OR (exclusion criteri*) OR review*[TIAB] OR search* OR analysis[TIAB] OR critique*[TIAB] OR appraisal OR "mantel haenszel" OR peto OR dersimonian OR (der simonian) OR handsearch*[TIAB] OR bibliography*[TIAB] OR citation* OR database*[TIAB] OR "medline" OR "embase" OR "scisearch" OR "science citation" OR "isi web" OR "web of science" OR internet[TIAB] OR reference* OR trial*[TIAB]))) NOT ("Case report"[TI] OR "editorial"[TI] OR "editorial"[PT] OR "letter"[PT] OR "newspaper article"[PT] OR "Clinical Trial"[PT])
3	#1 AND #2 Limits: English, French, Italian, Spanish, Catalan, Portuguese, All Adult: 19+ years, Publication Date from 2007 to 2011
2	("Depressive Disorder"[MH] OR "Depressive Disorder, Major"[MH] OR "Dysthymic Disorder"[MH] OR "Seasonal Affective Disorder"[MH] OR Depressi*[TI] OR Dysthym*[TI] OR Seasonal[TI] OR "Affective"[TI]) NOT

	("Bipolar Disorder"[MH] OR "Depression, Postpartum"[MH] OR Mania*[TI] OR Bipolar*[TI] OR Puerper*[TI] OR Postpart*[TI] OR Pregnan*[TI] OR Post-Natal*[TI] OR Postnatal[TI]) Limits: English, French, Italian, Spanish, Catalan, Portuguese, All Adult: 19+ years, Publication Date from 2007 to 2011
1	"Hypericum"[MeSH] OR "Hypericum perforatum" [TW] OR "Saint John's Wort" [TW] OR "St. John's Wort" [TW] OR "St. Johns Wort" [TW]

1.2.2. EMBASE

1	exp 'hypericum perforatum'/
2	("Hypericum" or "Hypericum perforatum" or "Saint John's Wort" or "St. John's Wort" or "St. Johns Wort").ti,sh,hw,ab,tn,dm,kw.
3	1 or 2
4	exp meta analysis/ or exp evidence based medicine/ or exp statistical analysis/
5	exp "meta analysis (topic)"/
6	(meta analysis or metaanalysis or meta-analysis).ti.
7	(meta analysis or metaanalysis or meta-analysis).ab.
8	"systematic review"/ or "systematic review (topic)"/
9	((systematic or literature) and review).ti.
10	((systematic or literature) and review).ab.
11	9 or 10
12	(evidence* or review* or overview* or survey or surveis).ab.
13	(evidence* or review* or overview* or survey or surveis).ti.
14	12 or 13
15	4 or 5 or 6 or 7 or 8 or 11 or 14
16	databases.ti. or databases.ab.
17	medline.ti. or medline.ab. or Embase.ti. or Embase.ab. or pubmed.ti. or Pubmed.ab. or Cocrhane.ti. or Cocrhane.ab. or Scisearch.ti. or Scisearch.ab. or Isi web.ti.
18	16 and 17
19	((handsearch and literature) or bibliograph*).ab.
20	18 or 19
21	15 and 20
22	exp depression/ or exp recurrent brief depression/ or exp.mp. [mp=title, abstract, subject headings, heading word, drug trade name, original title, device manufacturer, drug manufacturer, device trade name, keyword]
23	exp dysthymia/
24	exp seasonal affective disorder/
25	22 or 23 or 24
26	(depressi* or dysthym* or "Seasonal Affective" or Involutional*).ti.
27	exp manic psychosis/
28	exp bipolar disorder/
29	puerperal depression/
30	(Mania* or Bipolar* or Postpart* or Puerper* or Pregnant*).ti.
31	25 or 26
32	27 or 28 or 29 or 30

33	31 not 32
34	21 and 33
35	limit 34 to (embase and (basque or catalan or english or french or gallegan or italian or portuguese or spanish) and adult <18 to 64 years>)
36	3 and 35

1.2.3. Psychinfo

1	exp hypericum perforatum/
2	("Hypericum" or "Hypericum perforatum" or "Saint John's Wort" or "St. John's Wort" or "St. Johns Wort").ab,hw,id,sh,ti.
3	1 or 2
4	(metaanaly* or meta analy* or meta-analy*).tw.
5	(research or systematic or quantitative or methodologic*).af.
6	(overview* or review* or integration*).af.
7	5 and 6
8	databases.ti. or databases.ab.
9	medline.ti. or medline.ab. or Embase.ti. or Embase.ab. or pubmed.ti. or Pubmed.ab. or Cocrhane.ti. or Cocrhane.ab. or Scisearch.ti. or Scisearch.ab. or Isi web.ti.
10	((handsearch and literature) or bibliograph*).ab.
11	4 or 7
12	8 or 10
13	9 and 12
14	11 and 13
15	(case report or editorial).ti. or editorial.pt. or letter.ti. or letter.pt. or newspaper article.pt. or newspaper.ti.
16	14 not 15
17	exp seasonal affective disorder/
18	exp major depression/ or exp anaclitic depression/ or exp "depression (emotion)"/ or exp endogenous depression/ or exp reactive depression/ or exp recurrent depression/
19	depress*.ti.
20	seasonal affective disorder.ti.
21	17 or 18 or 19 or 20
22	exp postpartum depression/
23	exp pregnancy/
24	(mania* or bipolar* or pregnan* or postpart* or puerper*).ti.
25	exp bipolar disorder/
26	22 or 23 or 24 or 25
27	21 not 26
28	limit 27 to (adulthood <18+ years> and "300 adulthood " and (catalan or english or french or italian or portuguese or spanish) and yr="2007 - 2011")
29	16 and 28
30	3 and 29

2. *Restrictive approach*

2.1. Randomized controlled trials

2.1.1. Broad filter

9	(#8) AND (((((English[lang] OR French[lang] OR Italian[lang] OR Spanish[lang] OR Catalan[lang] OR Portuguese[lang])) AND ("2007/01/01"[PDat] : "2011/06/20"[PDat]))))
8	(Therapy/Broad[filter]) AND (#7)
7	(#3) AND #6
6	(#4) OR #5
5	John’s Wort[ti]
4	"Hypericum"[Mesh]
3	(#1) OR #2
2	Depress*[ti]
1	"Depressive Disorder"[Mesh]

2.1.2. Narrow filter

9	(#8) AND (((((English[lang] OR French[lang] OR Italian[lang] OR Spanish[lang] OR Catalan[lang] OR Portuguese[lang])) AND ("2007/01/01"[PDat] : "2011/06/20"[PDat]))))
8	(Therapy/Narrow[filter]) AND (#7)
7	(#3) AND #6
6	(#4) OR #5
5	John’s Wort[ti]
4	"Hypericum"[Mesh]
3	(#1) OR #2
2	Depress*[ti]
1	"Depressive Disorder"[Mesh]

2.2. Systematic reviews

	(#8) AND (((((English[lang] OR French[lang] OR Italian[lang] OR Spanish[lang] OR Catalan[lang] OR Portuguese[lang])) AND ("2007/01/01"[PDat] : "2011/06/20"[PDat]))))
	(#7) AND ((((((MEDLINE[Title/Abstract] OR (systematic[Title/Abstract] AND review[Title/Abstract]) OR meta analysis[Publication Type]))))))
	(#3) AND #6
	(#4) OR #5
	John’s Wort[ti]
	"Hypericum"[Mesh]
	(#1) OR #2
	Depress*[ti]
	"Depressive Disorder"[Mesh]

3. PLUS approach

((Depression [MeSH] ORDepressive Disorder[MeSH] ORDepressive Disorder, Major[MeSH] ORDepressive Disorder, Treatment-Resistant[MeSH] ORSuicide[MeSH] ORSuicidal Ideation[MeSH] ORSuicide, Assisted[MeSH] ORSuicide, Attempted[MeSH] ORAntidepressive Agents [MeSH] ORAntidepressive Agents, Second-Generation[MeSH] ORAntidepressive Agents, Tricyclic[MeSH]) OR (depressive disorder (disorder) [SNOMED CT: 35489007] OR depressive disorder (disorder) x Physical agent therapy (regime/therapy) [SNOMED CT: 35489007x229553000] OR depressive disorder (disorder) x Drug therapy (procedure) [SNOMED CT: 35489007x416608005] OR Depression(disorder) x psychological therapies (procedure) [SNOMED CT: 35489007x390822007] OR Self-injurious behavior (finding) [SNOMED CT: 248062006]))
