# Supplementary material for: Efficiency of pragmatic search strategies to update clinical guidelines recommendations
Source: BMC Med Res Methodol. 2015 Jul 31;15:57. doi: 10.1186/s12874-015-0058-2 (PMC4521498; doi:10.1186/s12874-015-0058-2)
Supplement: Additional file 2: — Key references by approach. We reported key references by strategy, according to their clinical guidelines and linked to recommendation. (PDF 62 kb) [file 12874_2015_58_MOESM2_ESM.pdf]

Additional file 2: Key references by approach

IdRef	CPG	IdRecom	Recommendation	IdDatabase	Key reference	Restrictive approach (narrow filter and clustering all questions) [0=No, 1=Yes]	PLUS approach [0=No, 1=Yes]
1	Major Depression in Adults 2008	1485	If the patient does not improve at the third or fourth week, any of the following strategies could be followed: 1)Switching from an antidepressant to any family, including another serotonergic. 2)Combining antidepressants. 3)Augmenting the initiated treatment with lithium or triiodothyronine.	17288688	Dorée JP, Des Rosiers J, Lew V, Gendron A, Elie R, Stip E, et al. Quetiapine augmentation of treatment-resistant depression: a comparison with lithium. Curr Med Res Opin. 2007;23(2):333-41.	1	0
2	Major Depression in Adults 2008	1485	If the patient does not improve at the third or fourth week, any of the following strategies could be followed: 1)Switching from an antidepressant to any family, including another serotonergic. 2)Combining antidepressants. 3)Augmenting the initiated treatment with lithium or triiodothyronine.	18047754	Cooper-Kazaz R, Lerer B. Efficacy and safety of triiodothyronine supplementation in patients with major depressive disorder treated with specific serotonin reuptake inhibitors. Int J Neuropsychopharmacol. 2008;11(5):685-99.	1	0
3	Major Depression in Adults 2008	1490	There is insufficient data for recommending augmentation with valproate, carbamazepine, lamotrigine, gabapentin or topiramate, pindolol, benzodiazepines, buspirone, methylphenidate or atypical antipsychotics.	17592905	Papakostas GI, Shelton RC, Smith J, Fava M. Augmentation of antidepressants with atypical antipsychotic medications for treatment-resistant major depressive disorder: a meta-analysis. J Clin Psychiatry. 2007;68(6):826-31.	1	0
4	Major Depression in Adults 2008	1490	There is insufficient data for recommending augmentation with valproate, carbamazepine, lamotrigine, gabapentin or topiramate, pindolol, benzodiazepines, buspirone, methylphenidate or atypical antipsychotics.	17592907	Berman RM, Marcus RN, Swanink R, McQuade RD, Carson WH, Corey-Lisle PK, et al. The efficacy and safety of aripiprazole as adjunctive therapy in major depressive disorder: a multicenter, randomized, double-blind, placebo-controlled study. J Clin Psychiatry. 2007;68(6):843-53.	1	0
5	Major Depression in Adults 2008	1490	There is insufficient data for recommending augmentation with valproate, carbamazepine, lamotrigine, gabapentin or topiramate, pindolol, benzodiazepines, buspirone, methylphenidate or atypical antipsychotics.	17975181	Mahmoud RA, Pandina GJ, Turkoz I, Kosik-Gonzalez C, Canuso CM, Kujawa MJ, et al. Risperidone for treatment-refractory major depressive disorder: a randomized trial. Ann Intern Med. 2007;147(9):593-602.	1	1

Additional file 2: Key references by approach

6	Major Depression in Adults 2008	1490	There is insufficient data for recommending augmentation with valproate, carbamazepine, lamotrigine, gabapentin or topiramate, pindolol, benzodiazepines, buspirone, methylphenidate or atypical antipsychotics.	18344725	Marcus RN, McQuade RD, Carson WH, Hennicken D, Fava M, Simon JS, et al. The efficacy and safety of aripiprazole as adjunctive therapy in major depressive disorder: a second multicenter, randomized, double-blind, placebo-controlled study. J Clin Psychopharmacol. 2008;28(2):156-65.	1	1
7	Major Depression in Adults 2008	1490	There is insufficient data for recommending augmentation with valproate, carbamazepine, lamotrigine, gabapentin or topiramate, pindolol, benzodiazepines, buspirone, methylphenidate or atypical antipsychotics.	18586273	Keitner GI, Garlow SJ, Ryan CE, Ninan PT, Solomon DA, Nemeroff CB, et al. A randomized, placebo-controlled trial of risperidone augmentation for patients with difficult-to-treat unipolar, non-psychotic major depression. J Psychiatr Res. 2009;43(3):205-14.	1	0
8	Major Depression in Adults 2008	1490	There is insufficient data for recommending augmentation with valproate, carbamazepine, lamotrigine, gabapentin or topiramate, pindolol, benzodiazepines, buspirone, methylphenidate or atypical antipsychotics.	EL KHALILI	El Khalili, N., Bauer, M., Datto, C., Earley, W., Astrom, M., and Eriksson, H. Pooled analysis of adjunctive extended release quetiapine fumarate (Quetiapine XR) in patients with major depressive disorder (MDD). European Psychiatry Conference: 17th European Psychiatric Association, EPA Congress. Lisbon, Portugal. 2009. 24(pp S637).	0	0
9	Major Depression in Adults 2008	1490	There is insufficient data for recommending augmentation with valproate, carbamazepine, lamotrigine, gabapentin or topiramate, pindolol, benzodiazepines, buspirone, methylphenidate or atypical antipsychotics.	19358791	Bauer M, Pretorius HW, Constant EL, Earley WR, Szamosi J, Brecher M. Extended-release quetiapine as adjunct to an antidepressant in patients with major depressive disorder: results of a randomized, placebo-controlled, double-blind study. J Clin Psychiatry. 2009;70(4):540-9.	1	0
10	Major Depression in Adults 2008	1490	There is insufficient data for recommending augmentation with valproate, carbamazepine, lamotrigine, gabapentin or topiramate, pindolol, benzodiazepines, buspirone, methylphenidate or atypical antipsychotics.	19687129	Nelson JC, Papakostas GI. Atypical antipsychotic augmentation in major depressive disorder: a meta-analysis of placebo-controlled randomized trials. Am J Psychiatry. 2009;166(9):980-91.	0	1
11	Major Depression in Adults 2008	1490	There is insufficient data for recommending augmentation with valproate, carbamazepine, lamotrigine, gabapentin or topiramate, pindolol, benzodiazepines, buspirone, methylphenidate or atypical antipsychotics.	20433324	Thomas SP, Nandhra HS, Jayaraman A. Systematic review of lamotrigine augmentation of treatment resistant unipolar depression (TRD). J Ment Health. 2010;19(2):168-75.	1	0

Additional file 2: Key references by approach

12	Major Depression in Adults 2008	1490	There is insufficient data for recommending augmentation with valproate, carbamazepine, lamotrigine, gabapentin or topiramate, pindolol, benzodiazepines, buspirone, methylphenidate or atypical antipsychotics.	20175941	El-Khalili N, Joyce M, Atkinson S, Buynak RJ, Datto C, Lindgren P, et al. Extended-release quetiapine fumarate (quetiapine XR) as adjunctive therapy in major depressive disorder (MDD) in patients with an inadequate response to ongoing antidepressant treatment: a multicentre, randomized, double-blind, placebo-controlled study. Int J Neuropsychopharmacol. 2010;13(7):917-32.	1	0
13	Major Depression in Adults 2008	1511	The use of St John's Wort is not recommended as a treatment option for patients with major depression.	18843608	Linde K, Berner MM, Kriston L. St John's wort for major depression. Cochrane Database Syst Rev. 2008 Oct 8;(4):CD000448. doi: 0.1002/14651858.CD000448.pub3.	0	1
14	Obesity in Childhood and Adolescence 2009	1050	Schools should include educational programmes which aim to improve diet, increase physical activity levels and reduce sedentary lifestyles. These should include families and teaching staff	21679476	Bjelland M, Bergh IH, Grydeland M, Klepp KI, Andersen LF, Anderssen SA, et al. Changes in adolescents' intake of sugar-sweetened beverages and sedentary behaviour: results at 8 month mid-way assessment of the HEIA study--a comprehensive, multi-component school-based randomized trial. Int J Behav Nutr Phys Act. 2011;8:63.	1	0
15	Obesity in Childhood and Adolescence 2009	1053	Multidisciplinary interventions should be implemented in schools to encourage children and adolescents to eat fruit and vegetables.	18719006	de Sa J, Lock K. Will European agricultural policy for school fruit and vegetables improve public health? A review of school fruit and vegetable programmes. Eur J Public Health. 2008;18(6):558-68.	1	0
16	Obesity in Childhood and Adolescence 2009	1053	Multidisciplinary interventions should be implemented in schools to encourage children and adolescents to eat fruit and vegetables.	21371499	Hoffman JA, Thompson DR, Franko DL, Power TJ, Leff SS, Stallings VA. Decaying behavioral effects in a randomized, multi-year fruit and vegetable intake intervention. Prev Med. 2011;52(5):370-5.	1	0
17	Obesity in Childhood and Adolescence 2009	1095	Physical activity programmes outside school hours are recommended for children and adolescents. These must be suited to their ages and preferences.	21852659	de Heer HD, Koehly L, Pederson R, Morera O. Effectiveness and spillover of an after-school health promotion program for Hispanic elementary school children. Am J Public Health. 2011;101(10):1907-13.	1	0
18	Obesity in Childhood and Adolescence 2009	1101	Educational programmes that target the family so as to encourage a healthy lifestyle are needed. These must cover healthy eating, education to understand nutritional information on food labels and the promotion of active leisure activities.	18180410	Paineau DL, Beaufils F, Boulier A, Cassuto DA, Chwalow J, Combris P, et al. Family dietary coaching to improve nutritional intakes and body weight control: a randomized controlled trial. Arch Pediatr Adolesc Med. 2008;162(1):34-43.	1	1
19	Obesity in Childhood and Adolescence 2009	1101	Educational programmes that target the family so as to encourage a healthy lifestyle are needed. These must cover healthy eating, education to understand nutritional information on food labels and the promotion of active leisure activities.	20107454	Olvera N, Bush JA, Sharma SV, Knox BB, Scherer RL, Butte NF. BOUNCE: a community-based mother-daughter healthy lifestyle intervention for low-income Latino families. Obesity (Silver Spring). 2010;18 **Suppl** 1:S102-4.	1	0

Additional file 2: Key references by approach

20	Obesity in Childhood and Adolescence 2009	1101	Educational programmes that target the family so as to encourage a healthy lifestyle are needed. These must cover healthy eating, education to understand nutritional information on food labels and the promotion of active leisure activities.	20107464	Fulkerson JA, Rydell S, Kubik MY, Lytle L, Boutelle K, Story M, et al. Healthy Home Offerings via the Mealtime Environment (HOME): feasibility, acceptability, and outcomes of a pilot study. Obesity (Silver Spring). 2010;18 **Suppl** 1:S69-74.	1	0
21	Obesity in Childhood and Adolescence 2009	1101	Educational programmes that target the family so as to encourage a healthy lifestyle are needed. These must cover healthy eating, education to understand nutritional information on food labels and the promotion of active leisure activities.	19933120	Chen JL, Weiss S, Heyman MB, Lustig RH. Efficacy of a child-centred and family-based program in promoting healthy weight and healthy behaviors in Chinese American children: a randomized controlled study. J Public Health (Oxf). 2010;32(2):219-29.	1	0
22	Obesity in Childhood and Adolescence 2009	1101	Educational programmes that target the family so as to encourage a healthy lifestyle are needed. These must cover healthy eating, education to understand nutritional information on food labels and the promotion of active leisure activities.	21041592	Robinson TN, Matheson DM, Kraemer HC, Wilson DM, Obarzanek E, Thompson NS, et al. A randomized controlled trial of culturally tailored dance and reducing screen time to prevent weight gain in low-income African American girls: Stanford GEMS. Arch Pediatr Adolesc Med. 2010;164(11):995-1004.	1	1
23	Obesity in Childhood and Adolescence 2009	1101	Educational programmes that target the family so as to encourage a healthy lifestyle are needed. These must cover healthy eating, education to understand nutritional information on food labels and the promotion of active leisure activities.	20508978	Karanja N, Lutz T, Ritenbaugh C, Maupome G, Jones J, Becker T, et al. The TOTS community intervention to prevent overweight in American Indian toddlers beginning at birth: a feasibility and efficacy study. J Community Health. 2010;35(6):667-75.	1	0
24	Obesity in Childhood and Adolescence 2009	1101	Educational programmes that target the family so as to encourage a healthy lifestyle are needed. These must cover healthy eating, education to understand nutritional information on food labels and the promotion of active leisure activities.	20576004	Monasta L, Batty GD, Macaluso A, Ronfani L, Lutje V, Bavcar A, et al. Interventions for the prevention of overweight and obesity in preschool children: a systematic review of randomized controlled trials. Obes Rev. 2011;12(5):e107-18.	1	0
25	Obesity in Childhood and Adolescence 2009	1119	Televisions, video consoles and computers should be removed from the bedrooms of children and adolescents who are overweight or obese.	21708797	Maniccia DM, Davison KK, Marshall SJ, Manganello JA, Dennison BA. A meta-analysis of interventions that target children's screen time for reduction. Pediatrics. 2011;128(1):e193-210.	1	0
26	Obesity in Childhood and Adolescence 2009	1125	Combined interventions including diet, physical exercise and changes to behaviour, with family involvement, are recommended for weight loss in children and adolescents aged 6-16 who are overweight or obese.	19160202	Oude Luttikhuis H, Baur L, Jansen H, Shrewsbury VA, O'Malley C, Stolk RP, et al. Interventions for treating obesity in children. Cochrane Database Syst Rev. 2009 Jan 21;(1):CD001872. doi: 10.1002/14651858.CD001872.pub2.		1

Additional file 2: Key references by approach

27	Obesity in Childhood and Adolescence 2009	1125	Combined interventions including diet, physical exercise and changes to behaviour, with family involvement, are recommended for weight loss in children and adolescents aged 6-16 who are overweight or obese.	19628106	Yackobovitch-Gavan M, Nagelberg N, Phillip M, Ashkenazi-Hoffnung L, Hershkovitz E, Shalitin S. The influence of diet and/or exercise and parental compliance on health-related quality of life in obese children. Nutr Res. 2009;29(6):397-404.		0
28	Obesity in Childhood and Adolescence 2009	1125	Combined interventions including diet, physical exercise and changes to behaviour, with family involvement, are recommended for weight loss in children and adolescents aged 6-16 who are overweight or obese.	Reinehr	Reinehr T, Schaefer A, Winkel K, Finne E, Kolip P. An effective lifestyle intervention in overweight children ('Obeldicks light'): Long-term findings from a randomized controlled trial. Obesity Reviews. 2010.		0
29	Obesity in Childhood and Adolescence 2009	1125	Combined interventions including diet, physical exercise and changes to behaviour, with family involvement, are recommended for weight loss in children and adolescents aged 6-16 who are overweight or obese.	20107463	Sacher PM, Kolotourou M, Chadwick PM, Cole TJ, Lawson MS, Lucas A, et al. Randomized controlled trial of the MEND program: a family-based community intervention for childhood obesity. Obesity (Silver Spring). 2010;18 **Suppl** 1:S62-8.		0
30	Obesity in Childhood and Adolescence 2009	1125	Combined interventions including diet, physical exercise and changes to behaviour, with family involvement, are recommended for weight loss in children and adolescents aged 6-16 who are overweight or obese.	20447648	Okely AD, Collins CE, Morgan PJ, Jones RA, Warren JM, Cliff DP, et al. Multi-site randomized controlled trial of a child-centered physical activity program, a parent-centered dietary-modification program, or both in overweight children: the HIKCUPS study. J Pediatr. 2010;157(3):388-94, 394.e1.		0
31	Obesity in Childhood and Adolescence 2009	1125	Combined interventions including diet, physical exercise and changes to behaviour, with family involvement, are recommended for weight loss in children and adolescents aged 6-16 who are overweight or obese.	20655544	Jelalian E, Lloyd-Richardson EE, Mehlenbeck RS, Hart CN, Flynn-O'Brien K, Kaplan J, et al. Behavioral weight control treatment with supervised exercise or peer-enhanced adventure for overweight adolescents. J Pediatr. 2010;157(6):923-928.e1.		1
32	Obesity in Childhood and Adolescence 2009	1125	Combined interventions including diet, physical exercise and changes to behaviour, with family involvement, are recommended for weight loss in children and adolescents aged 6-16 who are overweight or obese.	21135337	Ciampa PJ, Kumar D, Barkin SL, Sanders LM, Yin HS, Perrin EM, et al. Interventions aimed at decreasing obesity in children younger than 2 years: a systematic review. Arch Pediatr Adolesc Med. 2010;164(12):1098-104.		1
33	Obesity in Childhood and Adolescence 2009	1125	Combined interventions including diet, physical exercise and changes to behaviour, with family involvement, are recommended for weight loss in children and adolescents aged 6-16 who are overweight or obese.	20070541	Kelly KP, Kirschenbaum DS. Immersion treatment of childhood and adolescent obesity: the first review of a promising intervention. Obes Rev. 2011;12(1):37-49.		0

Additional file 2: Key references by approach

34	Obesity in Childhood and Adolescence 2009	1125	Combined interventions including diet, physical exercise and changes to behaviour, with family involvement, are recommended for weight loss in children and adolescents aged 6-16 who are overweight or obese.	21300674	Savoye M, Nowicka P, Shaw M, Yu S, Dziura J, Chavent G, et al. Long-term results of an obesity program in an ethnically diverse pediatric population. Pediatrics. 2011;127(3):402-10.		1
35	Obesity in Childhood and Adolescence 2009	1125	Combined interventions including diet, physical exercise and changes to behaviour, with family involvement, are recommended for weight loss in children and adolescents aged 6-16 who are overweight or obese.	21444600	Collins CE, Okely AD, Morgan PJ, Jones RA, Burrows TL, Cliff DP, et al. Parent diet modification, child activity, or both in obese children: an RCT. Pediatrics. 2011 ;127(4):619-27.		0
36	Obesity in Childhood and Adolescence 2009	1125	Combined interventions including diet, physical exercise and changes to behaviour, with family involvement, are recommended for weight loss in children and adolescents aged 6-16 who are overweight or obese.	21487425	Coppins DF, Margetts BM, Fa JL, Brown M, Garrett F, Huelin S. Effectiveness of a multi-disciplinary family-based programme for treating childhood obesity (the Family Project). Eur J Clin Nutr. 2011;65(8):903-9.		0
37	Obesity in Childhood and Adolescence 2009	1125	Combined interventions including diet, physical exercise and changes to behaviour, with family involvement, are recommended for weight loss in children and adolescents aged 6-16 who are overweight or obese.	21514017	Schaefer A, Winkel K, Finne E, Kolip P, Reinehr T. An effective lifestyle intervention in overweight children: one-year follow-up after the randomized controlled trial on "Obeldicks light". Clin Nutr. 2011;30(5):629-33.		0
38	Obesity in Childhood and Adolescence 2009	1126	The clinical and family environments are the most appropriate settings for combined interventions for weight loss in children and adolescents who are overweight or obese.	20102858	Díaz RG, Esparza-Romero J, Moya-Camarena SY, Robles-Sardín AE, Valencia ME. Lifestyle intervention in primary care settings improves obesity parameters among Mexican youth. J Am Diet Assoc. 2010;110(2):285-90.		0
39	Obesity in Childhood and Adolescence 2009	1126	The clinical and family environments are the most appropriate settings for combined interventions for weight loss in children and adolescents who are overweight or obese.	20585269	Ellis DA, Janisse H, Naar-King S, Kolmodin K, Jen KL, Cunningham P, et al. The effects of multisystemic therapy on family support for weight loss among obese African-American adolescents: findings from a randomized controlled trial. J Dev Behav Pediatr. 2010;31(6):461-8.		0
40	Obesity in Childhood and Adolescence 2009	1126	The clinical and family environments are the most appropriate settings for combined interventions for weight loss in children and adolescents who are overweight or obese.	21262890	Magarey AM, Perry RA, Baur LA, Steinbeck KS, Sawyer M, Hills AP, et al. A parent-led family-focused treatment program for overweight children aged 5 to 9 years: the PEACH RCT. Pediatrics. 2011;127(2):214-22.		1

Additional file 2: Key references by approach

41	Obesity in Childhood and Adolescence 2009	1126	The clinical and family environments are the most appropriate settings for combined interventions for weight loss in children and adolescents who are overweight or obese.	21487425	Coppins DF, Margetts BM, Fa JL, Brown M, Garrett F, Huelin S. Effectiveness of a multi-disciplinary family-based programme for treating childhood obesity (the Family Project). Eur J Clin Nutr. 2011;65(8):903-9.		0
42	Obesity in Childhood and Adolescence 2009	1129	For adolescents (aged 12-18) suffering from obesity and severe comorbidities who have not responded to dietary and lifestyle treatment, orlistat* treatment (120 mg with breakfast, lunch and dinner) may be considered as part of a programme of changes to lifestyle. This must be supervised by specialists in endocrinology and nutrition, family medicine or paediatrics who have been trained to treat obesity. * Sibutramine and orlistat are not funded by Spanish Social Security.	19160202	Oude Luttikhuis H, Baur L, Jansen H, Shrewsbury VA, O'Malley C, Stolk RP, et al. Interventions for treating obesity in children. Cochrane Database Syst Rev. 2009 Jan 21;(1):CD001872. doi: 10.1002/14651858.CD001872.pub2.	1	1
43	Obesity in Childhood and Adolescence 2009	1129	For adolescents (aged 12-18) suffering from obesity and severe comorbidities who have not responded to dietary and lifestyle treatment, orlistat* treatment (120 mg with breakfast, lunch and dinner) may be considered as part of a programme of changes to lifestyle. This must be supervised by specialists in endocrinology and nutrition, family medicine or paediatrics who have been trained to treat obesity. * Sibutramine and orlistat are not funded by Spanish Social Security.	20083531	Whitlock EP, O'Connor EA, Williams SB, Beil TL, Lutz KW. Effectiveness of weight management interventions in children: a targeted systematic review for the USPSTF. Pediatrics. 2010;125(2):e396-418.	1	1
44	Obesity in Childhood and Adolescence 2009	1129	For adolescents (aged 12-18) suffering from obesity and severe comorbidities who have not responded to dietary and lifestyle treatment, orlistat* treatment (120 mg with breakfast, lunch and dinner) may be considered as part of a programme of changes to lifestyle. This must be supervised by specialists in endocrinology and nutrition, family medicine or paediatrics who have been trained to treat obesity. * Sibutramine and orlistat are not funded by Spanish Social Security.	19922432	Viner RM, Hsia Y, Tomsic T, Wong IC. Efficacy and safety of anti-obesity drugs in children and adolescents: systematic review and meta-analysis. Obes Rev. 2010;11(8):593-602.	1	0

Additional file 2: Key references by approach

45	Obesity in Childhood and Adolescence 2009	1129	For adolescents (aged 12-18) suffering from obesity and severe comorbidities who have not responded to dietary and lifestyle treatment, orlistat* treatment (120 mg with breakfast, lunch and dinner) may be considered as part of a programme of changes to lifestyle. This must be supervised by specialists in endocrinology and nutrition, family medicine or paediatrics who have been trained to treat obesity. * Sibutramine and orlistat are not funded by Spanish Social Security.	20858149	Chanoine JP, Richard M. Early weight loss and outcome at one year in obese adolescents treated with orlistat or placebo. Int J Pediatr Obes. 2011;6(2):95-101.	1	0
46	Prostate Cancer Treatment 2008	1204	In patients with prostate cancer at a locally advanced stage with a life expectancy less than 10 years, watching and waiting or hormone therapy may be therapeutic alternatives.	18349064	Shahani S, Braga-Basaria M, Basaria S. Androgen deprivation therapy in prostate cancer and metabolic risk for atherosclerosis. J Clin Endocrinol Metab. 2008;93(6):2042-9.		0
47	Prostate Cancer Treatment 2008	1206	The normal duration of neoadjuvant hormonal treatment with radiotherapy in patients with prostate cancer at a locally advanced stage is 3 months.	21440505	Denham JW, Steigler A, Lamb DS, Joseph D, Turner S, Matthews J, et al. Short-term neoadjuvant androgen deprivation and radiotherapy for locally advanced prostate cancer: 10-year data from the TROG 96.01 randomised trial. Lancet Oncol. 2011;12(5):451-9.		1
48	Prostate Cancer Treatment 2008	1208	The normal duration of neoadjuvant hormonal treatment after radiotherapy in patients with prostate cancer at a locally advanced stage is 2-3 years.	17102802	Wirth M, Tyrrell C, Delaere K, Sánchez-Chapado M, Ramon J, Wallace DM, et al. Bicalutamide (Casodex) 150 mg plus standard care in early non-metastatic prostate cancer: results from Early Prostate Cancer Trial 24 at a median 7 years' follow-up. Prostate Cancer Prostatic Dis. 2007;10(1):87-93.		0
49	Prostate Cancer Treatment 2008	1208	The normal duration of neoadjuvant hormonal treatment after radiotherapy in patients with prostate cancer at a locally advanced stage is 2-3 years.	MARTINEZ-PINEIRO	Martinez-Pineiro L. Does neoadjuvant and adjuvant treatment improve outcome in localised prostatic cancer? European Urology Supplements. 2008;7(5):406-409.		0
50	Prostate Cancer Treatment 2008	1208	The normal duration of neoadjuvant hormonal treatment after radiotherapy in patients with prostate cancer at a locally advanced stage is 2-3 years.	COLLETTE	Collette L, Mauer M, Bolla M, Van Tienhoven G, De Reijke TM, Van Den Bergh ACM, et al. 745 health related quality of life and symptoms in an international phase iii trial of long term versus short-term androgen suppression and radiation therapy for locally advanced prostate cancer (eortc trial 22961). European Urology Supplements. 2009;8(4):307.		0

Additional file 2: Key references by approach

51	Prostate Cancer Treatment 2008	1208	The normal duration of neoadjuvant hormonal treatment after radiotherapy in patients with prostate cancer at a locally advanced stage is 2-3 years.	BOLLA	Bolla M, Collette L, Van Tienhoven G, Warde P, Dubois JB, Mirimanoff RO, et al. 7007 Three years of adjuvant androgen deprivation with goserelin in patients with locally advanced prostate cancer treated with radiotherapy: Results at 10 years of EORTC trial 22863. European Journal of Cancer Supplements. 2009;7(2):408.		0
52	Prostate Cancer Treatment 2008	1208	The normal duration of neoadjuvant hormonal treatment after radiotherapy in patients with prostate cancer at a locally advanced stage is 2-3 years.	22129214	Iversen P, McLeod DG, See WA, Morris T, Armstrong J, Wirth MP, et al. Antiandrogen monotherapy in patients with localized or locally advanced prostate cancer: final results from the bicalutamide Early Prostate Cancer programme at a median follow-up of 9.7 years. BJU Int. 2010;105(8):1074-81.		0
53	Prostate Cancer Treatment 2008	1230	In patients with disseminated prostate cancer for whom hormone therapy has been indicated, castration (surgical or chemical) is recommended as a first-line treatment.	18491137	Arai Y, Akaza H, Deguchi T, Fujisawa M, Hayashi M, Hirao Y, et al. Evaluation of quality of life in patients with previously untreated advanced prostate cancer receiving maximum androgen blockade therapy or LHRHa monotherapy: a multicenter, randomized, double-blind, comparative study. J Cancer Res Clin Oncol. 2008;134(12):1385-96.	1	0
54	Prostate Cancer Treatment 2008	1233	In patients with disseminated prostate cancer and low tumour load, intermittent androgen suppression may be assessed as an alternative to continuous androgen suppression if there is a good response to the initial hormone treatment.	19249153	Calais da Silva FE, Bono AV, Whelan P, Brausi M, Marques Queimadelos A, Martin JA, et al. Intermittent androgen deprivation for locally advanced and metastatic prostate cancer: results from a randomized phase 3 study of the South European Uroncological Group. Eur Urol. 2009;55(6):1269-77.	1	0
55	Prostate Cancer Treatment 2008	1234	To be able to indicate intermittent hormone therapy, the patient must have received androgen deprivation for at least 7 months and reached a PSA < 4 ng/ml (stable or in decline during the sixth and seventh months), or a 90% reduction from pre-treatment levels. Monitoring will be carried out every 6 months. Patients who have stopped androgen deprivation will receive another cycle on request, when the PSA increases or when clinical symptoms of disease progression appear. If the PSA returns to normal after the new round of androgen deprivation, hormone therapy can be stopped again.	19249153	Calais da Silva FE, Bono AV, Whelan P, Brausi M, Marques Queimadelos A, Martin JA, et al. Intermittent androgen deprivation for locally advanced and metastatic prostate cancer: results from a randomized phase 3 study of the South European Uroncological Group. Eur Urol. 2009;55(6):1269-77.	1	0

Additional file 2: Key references by approach

56	Prostate Cancer Treatment 2008	1243	The systematic use of bisphosphonates (zoledronic acid) as a preventive treatment in bone complications is not recommended. Zoledronic acid (4 mg every 3 weeks) can be offered in selected hormone-independent patients with demonstrated metastasis.	21353695	Fizazi K, Carducci M, Smith M, Damião R, Brown J, Karsh L, et al. Denosumab versus zoledronic acid for treatment of bone metastases in men with castration-resistant prostate cancer: a randomised, double-blind study. Lancet. 2011;377(9768):813-22.	1	0
57	Secondary Prevention of Stroke 2009	1400	Patients who have suffered a stroke are encouraged to exercise regularly within their capabilities and reduce body weight or abdominal obesity to normal levels.	19821305	Saunders DH, Greig CA, Mead GE, Young A. Physical fitness training for stroke patients. Cochrane Database Syst Rev. 2009 Oct 7;(4):CD003316. doi: 10.1002/14651858.CD003316.pub3.	0	0
58	Secondary Prevention of Stroke 2009	1400	Patients who have suffered a stroke are encouraged to exercise regularly within their capabilities and reduce body weight or abdominal obesity to normal levels.	22071806	Brazzelli M, Saunders DH, Greig CA, Mead GE. Physical fitness training for stroke patients. Cochrane Database Syst Rev. 2011 Nov 9;(11):CD003316. doi: 10.1002/14651858.CD003316.pub4.	0	0
59	Secondary Prevention of Stroke 2009	1406	It is recommended to treat patients with ischemic stroke or prior transient ischemic attack of atherothrombotic etiology with atorvastatin (80 mg/d), regardless of their basal LDL-cholesterol levels.	19228842	Amarenco P, Benavente O, Goldstein LB, Callahan A 3rd, Sillesen H, Hennerici MG, et al. Results of the Stroke Prevention by Aggressive Reduction in Cholesterol Levels (SPARCL) trial by stroke subtypes. Stroke. 2009;40(4):1405-9.	1	0
60	Secondary Prevention of Stroke 2009	1406	It is recommended to treat patients with ischemic stroke or prior transient ischemic attack of atherothrombotic etiology with atorvastatin (80 mg/d), regardless of their basal LDL-cholesterol levels.	19588332	Manktelow BN, Potter JF. Interventions in the management of serum lipids for preventing stroke recurrence. Cochrane Database Syst Rev. 2009 Jul 8;(3):CD002091. doi: 10.1002/14651858.CD002091.pub2.	1	1
61	Secondary Prevention of Stroke 2009	1407	Treatment with other statins (simvastatin 40 mg) is also indicated in patients with ischemic stroke or prior transient ischemic attack of atherothrombotic etiology, regardless of their basal LDL-cholesterol levels.	17986516	Henyan NN, Riche DM, East HE, Gann PN. Impact of statins on risk of stroke: a meta-analysis. Ann Pharmacother. 2007;41(12):1937-45.	1	0
62	Secondary Prevention of Stroke 2009	1407	Treatment with other statins (simvastatin 40 mg) is also indicated in patients with ischemic stroke or prior transient ischemic attack of atherothrombotic etiology, regardless of their basal LDL-cholesterol levels.	19588332	Manktelow BN, Potter JF. Interventions in the management of serum lipids for preventing stroke recurrence. Cochrane Database Syst Rev. 2009 Jul 8;(3):CD002091. doi: 10.1002/14651858.CD002091.pub2.	1	1
63	Secondary Prevention of Stroke 2009	1410	The combination of statins with other hypolipemiant drugs to reach LDLcholesterol target values should be avoided.	19884623	Sharma M, Ansari MT, Abou-Setta AM, Soares-Weiser K, Ooi TC, Sears M, et al. Systematic review: comparative effectiveness and harms of combination therapy and monotherapy for dyslipidemia. Ann Intern Med. 2009;151(9):622-30.	1	0

Additional file 2: Key references by approach

64	Secondary Prevention of Stroke 2009	1417	In cases where anticoagulant treatment is contraindicated, treatment with aspirin (300 mg/d) is an appropriate alternative.	19336502	ACTIVE Investigators, Connolly SJ, Pogue J, Hart RG, Hohnloser SH, Pfeffer M, et al. Effect of clopidogrel added to aspirin in patients with atrial fibrillation. N Engl J Med. 2009;360(20):2066-78.	1	1
65	Secondary Prevention of Stroke 2009	1417	In cases where anticoagulant treatment is contraindicated, treatment with aspirin (300 mg/d) is an appropriate alternative.	20388864	Ansara AJ, Nisly SA, Arif SA, Koehler JM, Nordmeyer ST. Aspirin dosing for the prevention and treatment of ischemic stroke: an indication-specific review of the literature. Ann Pharmacother. 2010;44(5):851-62.	1	0
66	Secondary Prevention of Stroke 2009	1417	In cases where anticoagulant treatment is contraindicated, treatment with aspirin (300 mg/d) is an appropriate alternative.	21309657	Connolly SJ, Eikelboom J, Joyner C, Diener HC, Hart R, Golitsyn S, et al. Apixaban in patients with atrial fibrillation. N Engl J Med. 2011;364(9):806-17.	1	1
67	Secondary Prevention of Stroke 2009	1417	In cases where anticoagulant treatment is contraindicated, treatment with aspirin (300 mg/d) is an appropriate alternative.	HART	Hart RG, Eikelboom J, Yusuf S, Gao P, Paolasso E, De Caterina R, et al. Efficacy and safety of the novel oral factor Xa inhibitor apixaban in atrial fibrillation (AF) patients with chronic kidney disease (CKD): The AVERROES trial. European Heart Journal. Conference: European Society of Cardiology, ESC Congress. 2011.	0	0
68	Secondary Prevention of Stroke 2009	1424	In patients with previous ischemic stroke or transient ischemic attack who present permeable foramen ovale, treatment with antiaggregants (100-300 mg/d of aspirin) is recommended.	KENT	Kent DM, Kitsios GD. Meta-analysis of observational studies for the risk of recurrent stroke with PFO closure or medical treatment and comparison to the results of the closure I trial. Cerebrovascular Diseases. Conference. 2011; Hamburg, Germany.	0	0
69	Secondary Prevention of Stroke 2009	1427	Carotid endarterectomy is recommended in patients with ischemic stroke of less than 6 months evolution and significant stenosis of the carotid artery (70% to 99%, NASCET values), if and when the surgical team confirms a perioperative morbimortality of less than 6%.	19769803	Patterson BO, Holt PJ, Hinchliffe RJ, Thompson MM, Loftus IM. Urgent carotid endarterectomy for patients with unstable symptoms: systematic review and meta-analysis of outcomes. Vascular. 2009;17(5):243-52.	1	0
