# Supplementary material for: Efficiency of pragmatic search strategies to update clinical guidelines recommendations
Source: BMC Med Res Methodol. 2015 Jul 31;15:57. doi: 10.1186/s12874-015-0058-2 (PMC4521498; doi:10.1186/s12874-015-0058-2)
Supplement: Additional file 3: — Additional tables. We reported complementary results (PDF 40 kb) [file 12874_2015_58_MOESM3_ESM.pdf]

Additional file 3: Additional tables

Restrictive approach (narrow filter and clustering all questions) recommendations results

	Recommendations not identify by restrictive approach (n=3)		Recommendations identify by restrictive approach (n=17)		p*
**CPGs topic, n (%)**					
. Mental health	1	33,3	2	11,8	0,312
. Metabolic disease	0	0,0	6	35,3	
. Cancer and palliative care	0	0,0	4	23,5	
. Cardiovascular disease	2	66,7	5	29,4	
**Strength of recommendations (SIGN system), n (%)**					
. A	0	0,0	3	17,6	0,151
. B	3	100,0	5	29,4	
. C	0	0,0	2	11,8	
. D	-	-	-	-	
. GPP	0	0,0	7	41,2	
**Section purpose, n (%)**					
. Prevention	2	66,7	11	64,7	1,000
. Screening	-	-	-	-	
. Treatment	1	33,3	6	35,3	
. Others	-	-	-	-	
**Recommendation turnover, n (%)**					
. Without references	-	-	-	-	1,000
. With low references	1	33,3	5	29,4	
. With high references	2	66,7	12	70,6	
**Total**	3		17		

Abbreviations: CPG: Clinical practice guideline; GPP: Good practice point, ReSe:

*Pearson's chi-square test or Fisher's Exact Test, as appropiate.

Additional file 3: Additional tables

PLUS approach recommendations results

	Recommendations not identify by PLUS approach (n=15)		Recommendations identify by PLUS approach (n=10)		p*
**CPGs topic, n (%)**					
. Mental health	1	6,7	2	20,0	0,365
. Metabolic disease	4	26,7	4	40,0	
. Cancer and palliative care	6	40,0	1	10,0	
. Cardiovascular disease	4	26,7	3	30,0	
**Strength of recommendations (SIGN system), n (%)**					
. A	2	13,3	1	10,0	0,763
. B	6	40,0	3	30,0	
. C	1	6,7	2	20,0	
. D	1	6,7	0	0,0	
. GPP	5	33,3	4	40,0	
**Section purpose, n (%)**					
. Prevention	8	53,3	7	70,0	0,678
. Screening	-	-	-	-	
. Treatment	7	46,7	3	30,0	
. Others	-	-	-	-	
**Recommendation turnover, n (%)**					
. Without references	-	-	-	-	0,020
. With low references	7	46,7	0	0,0	
. With high references	8	53,3	10	100,0	
**Total**	15		10		
Abbreviations: CPG: Clinical practice guideline; GPP: Good practice point.					
*Pearson's chi-square test or Fisher's Exact Test, as appropiate.					
